# Cooled Radiofrequency Ablation for Intercostal Neuralgia

**DOI:** 10.31486/toj.22.0087

**Published:** 2023

**Authors:** Kenneth J. Fiala, Joshua M. Martens, Mitchell K. Keith, Adam Ghouse, Alaa Abd-Elsayed

**Affiliations:** ^1^ University of Wisconsin-Madison School of Medicine and Public Health, Madison, WI; ^2^ Department of Anesthesiology, University of Wisconsin-Madison School of Medicine and Public Health, Madison, WI

**Keywords:** *Chronic pain*, *intercostal nerves*, *neuralgia*, *radiofrequency ablation*, *radiofrequency therapy*

## Abstract

**Background:** Intercostal neuralgia is pain associated with the intercostal nerves along the rib, chest, and upper abdominal wall. Intercostal neuralgia has various etiologies, and current conventional treatment options include intercostal nerve blocks, nonsteroidal anti-inflammatory drugs, transcutaneous electrical nerve stimulation, topical medications, opioids, tricyclic antidepressants, and anticonvulsants. For a subset of patients, these conventional treatment options provide little relief. Radiofrequency ablation (RFA) is an emerging procedure for the treatment of chronic pain and neuralgias. Cooled RFA (CRFA) is a specific type of RFA that has been trialed as a treatment for intercostal neuralgia in patients refractory to conventional treatment modalities. This case series assesses the efficacy of CRFA for the treatment of intercostal neuralgia in 6 patients.

**Case Series:** Three female and 3 male patients underwent CRFA of the intercostal nerves to treat intercostal neuralgia. The patients had an average age of 50.7 years and demonstrated an average pain reduction of 81.3%.

**Conclusion:** This case series suggests that CRFA may be an effective treatment option for patients with intercostal neuralgia that is not responsive to conservative treatment options. To determine the duration of pain improvement, large research studies need to be conducted.

## INTRODUCTION

Intercostal neuralgia is any painful condition of the intercostal nerves along the rib, chest, and upper abdominal wall^1^ that presents as a type of neuropathic pain characterized as sharp, shooting, or burning.^2^ The chronic pain of intercostal neuralgia impacts patients’ activities of daily living, inhibiting breathing and movements.^3^ Intercostal neuralgia arises when an intercostal nerve supplying a portion of the chest wall and abdomen is damaged. Intercostal nerves arise from the anterior rami of the thoracic spinal nerves.^4^ Each nerve enters the appropriate intercostal space between the posterior intercostal membrane and the parietal pleura.^4^ After entering the intercostal space, the nerve advances to the subcostal groove of the rib.^4^ Causes of intercostal neuralgia include postoperative complications, traumatic injuries, neoplasms or postirradiation tumors, inflammation, chest tube placement, mastectomy, and infection of an intercostal nerve.^1,5-9^ Most commonly, intercostal neuralgia arises from a previous operation or from infection of an intercostal nerve.^1^

Generalized thoracic pain is prevalent in about 15% of the population, and approximately 40% of patients experience postthoracotomy pain.^3^ While the proportion of individuals suffering from abdominal pain is not known, females are up to 4 times more likely than males to suffer from chronic abdominal wall pain.^3^ Intercostal neuralgia is an umbrella term, so its specific prevalence cannot easily be determined.^1^

Conventional pain management modalities used with intercostal neuralgia include nonsteroidal anti-inflammatory drugs (NSAIDs);^10^ topical medications; opioids; anti-depressants;^11^ anticonvulsants;^12,13^ transcutaneous electrical nerve stimulation; and thoracic, epidural, and intercostal nerve blockade.^5^ However, these treatments have variable consequences and many patients do not experience significant, lasting reductions in their pain. Side effects associated with selective serotonin reuptake inhibitors are generally mild but include weight gain and sexual dysfunction.^11^ Side effects associated with tricyclic antidepressants include anticholinergic effects, orthostatic hypotension, and cardiovascular effects.^11^ Nausea, vomiting, constipation, physical dependance, tolerance, dizziness, sedation, and respiratory depression are all common side effects of opioids.^14^ Rapid absorption of local anesthetic with intercostal nerve blockades can lead to high systemic blood levels.^6^

The discovery and development of alternate treatments for intercostal neuralgia are essential to improve patient outcomes. Radiofrequency ablation (RFA) uses radiofrequency energy to create heat to achieve tissue necrosis.^15^ In this minimally invasive procedure, radio waves with a frequency of 100,000 to 500,000 Hz are applied near a target nerve with the intent to alter the nociceptive pathways of the nerve and thereby lessen the patient's pain.^16,17^ Cooled RFA (CRFA) is similar to conventional RFA, but in CRFA, water circulated inside the interior of the single-needle electrode tip^18^ intermittently cools the electrode tip. The goal tissue temperature of both RFA and CRFA is 80 °C; however, the device temperature is set to 60 °C for the CRFA procedure and 80 °C for the conventional RFA procedure. The lower temperature in CRFA helps prevent blood coagulation and tissue charring, keeping the pathway of the electrode clear for larger ablations than obtained with conventional RFA.^19^ When needed, multiple electrodes can be used during CRFA for larger treatment areas.^18^ Compared to the lesions produced with commercially available noncooled electrode tips, CRFA generates larger lesions.^20^

RFA has been demonstrated to successfully treat resistant intercostal neuralgia.^2^ Because CRFA generates a larger lesion than conventional RFA, we hypothesized that CRFA may be an efficacious treatment option for patients with intercostal neuralgia. This case series describes the efficacy of CRFA treatment for 6 patients with intercostal neuralgia refractory to conventional treatment measures. This case series underwent institutional review board review and received an exemption.

## PROCEDURE

After informed consent and preprocedure vitals were obtained, patients were placed in the prone position. The thoracic area of the target intercostal nerves was prepped with chlorhexidine and draped in sterile fashion. Lidocaine 1% was injected as local skin wheal anesthesia. Under fluoroscopic guidance, a 17-gauge, 50-mm needle with a 4-mm active tip was inserted and advanced until the needle was in contact with the inferior border of the target rib. The needle was then walked off the rib to just underneath the inferior edge. The same needle insertion was replicated for all target intercostal nerve spaces. RFA probes were inserted into each needle, and motor testing was performed for confirmation of placement. Each RFA probe was injected with 1 mL of lidocaine 2%. In lesion mode, CRFA was performed for 165 seconds to the tissue with a machine setpoint of 60 °C and a target tissue temperature of 80 °C around the electrode tip. After the procedure duration, the needles were removed, the skin was cleaned, and sterile dressing was applied.

## CASE SERIES

All of the patients who underwent CRFA of the intercostal nerves met our defined criteria for intercostal neuralgia refractory to conservative management. Patients were eligible for intervention if they experienced chronic rib, chest, or upper abdominal wall pain attributable to intercostal neuralgia and did not experience adequate relief following conservative management with a combination of pharmacotherapy, physical therapy, heat, ice, and rest. All 6 patients received preprocedural diagnostic intercostal nerve blocks. None of the patients reported any increase in pain during the procedure, and all 6 patients tolerated the procedure well.

### Case 1

A 72-year-old female with a history of hypertension, lumbar spondylosis, osteoarthritis of the lumbar spine, osteoporosis, and thoracic spondylosis presented to the interventional pain clinic with left-sided thoracic back pain. She rated the pain as 9/10 on the visual analog scale (VAS) prior to undergoing any procedural interventions. The patient had a known L2 vertebral body compression fracture and multilevel lumbar degenerative disc disease at the time of the procedure. The patient had received minimal relief with pharmacologic measures and lumbar level nerve blocks. On April 24, 2019, the patient underwent conventional RFA of the intercostal nerves at the T8-T11 levels but experienced little relief.

On February 12, 2020, the patient underwent CRFA of the left intercostal nerve at the levels of T8, T9, T10, and T11 under fluoroscopic guidance. One hundred four days after the procedure, the patient reported 100% improvement in her pain, improving from 9/10 to 0/10, but she reported a new aching, burning, left upper quadrant abdominal pain following the procedure. This developed pain could potentially be peripheral neuritis as a side effect from the CRFA therapy; however, the treatment and the pain are not believed to be linked because the pain was located at a different site than the treatment area, did not overlie sites of RFA intervention, and did not radiate. Since the 104-day post-CRFA follow-up, the patient has not mentioned a change in or return of her thoracic pain.

### Case 2

A 31-year-old female with a history of right upper quadrant abdominal pain and mild upper thoracic degenerative disc narrowing presented to the interventional pain clinic with right midback pain. She described the pain as a constant dull ache that radiated to her abdominal right upper quadrant that worsened with coughs and deep breaths. The patient rated the pain as 7/10 on the VAS and said it was tingling in nature. The patient received minimal relief with pharmacologic measures.

On May 13, 2020, the patient underwent CRFA of the right intercostal nerve at the levels of T7, T8, T9, and T10 under fluoroscopic guidance. Thirty-five days after the procedure, the patient reported 100% improvement of her pain, improving to 0/10 on the VAS. The patient did not report any negative symptoms. Since the 35-day post-CRFA follow-up, the patient has not mentioned a return of or change in her pain.

### Case 3

A 44-year-old male with a history of depression, costochondritis, lumbar facet neuralgia, and intercostal neuralgia presented to the interventional pain clinic with right upper chest and back pain. He rated the pain as 6/10 on the VAS and described it as achy, stabbing in nature, and intermittent. The patient reported that the pain improved with rest and worsened with activity. Pharmacologic and conservative measures provided minimal relief. This patient had received costochondral and trigger point injections from providers outside of the pain clinic. He also underwent right T1-T4 intercostal RFA 16 months prior that he said provided >70% relief. However, the pain returned after 12 months, and the patient re-presented to the pain clinic with chest and back pain.

On July 8, 2020, the patient underwent CRFA of the right intercostal nerves at the levels of T1, T2, T3, and T4 under fluoroscopic guidance. Two hundred fifty days following the procedure, the patient reported his pain as 2/10 on the VAS. He did not report any negative side effects from the procedure. Since the 250-day post-CRFA follow-up, the patient has not reported a return of or change in his pain.

### Case 4

A 54-year-old male with a history of hypertension, hyperlipidemia, obesity, complex regional pain syndrome, and a compression fracture of the T12 vertebra presented to the interventional pain clinic with right midback pain. The pain radiated around the chest and had been present since the patient fell at work 7 years prior to presentation. The patient rated the pain as 9/10 on the VAS and described it as achy in nature. The patient reported that the pain improved when he lay on his right side and applied ice and heat and worsened with speaking and movement. Pharmacologic and conservative measures did not provide significant relief. The patient had undergone right upper cervical paraspinal trigger point injection; right thoracolumbar paraspinal trigger point injection; right upper trapezius muscle trigger point injections; right costal facet joint injections (T10-T12); denervation of right costal facets with RFA (T7-T9); right C5-C7 RFA; and right C5-C6 transforaminal epidural steroid injection. The patient underwent 2 diagnostic right intercostal nerve blocks prior to CRFA at the levels of T9-T12 that resulted in good relief.

On November 11, 2020, the patient underwent CRFA of the right intercostal nerves at the levels of T9, T10, T11, and T12 under fluoroscopic guidance. This patient reported 90% pain relief lasting 334 days and did not mention any complaints about the procedure.

### Case 5

A 46-year-old female with a history of migraine headaches, anxiety, depression, xiphoidalgia syndrome, and costochondral junction syndrome presented to the interventional pain clinic with constant epigastric pain. She rated the pain as 5/10 on the VAS with intermittent episodes of shooting pain that reached 10/10. She described the pain as aching in nature and referring from her back to her sternum in a band-shape distribution over her ribs. She reported that the pain worsened with daily activities, talking on the phone, and sitting. The patient received insignificant relief with pharmacologic and conservative measures. She had undergone intercostal nerve blocks that provided moderate, temporary relief from the pain.

On November 25, 2020, the patient underwent CRFA of the left intercostal nerves at the levels of T9, T10, T11, and T12 under fluoroscopic guidance. Fifty days after the procedure, the patient reported 75% improvement in her pain but noted that the pain relief provided by CRFA was not immediate; the 75% relief was reached after 4 weeks. Because the pain relief was not 100%, the remaining pain was constant, and the patient continued to experience worsened pain with daily activities, talking on the phone, and sitting. She denied any other negative effects from the procedure. The patient's pain fully returned 7 months after the procedure.

### Case 6

A 57-year-old male with a history of hypertension and testosterone deficiency presented to the interventional pain clinic with a diagnosis of intercostal neuralgia at the level of T9 and T10 following a robotic surgery and chest tube placement for thoracic outlet syndrome. He rated the pain as 7/10 on the VAS and displayed 2-point discrimination. The pain referred from the left lower chest around to the abdomen and was associated with allodynia. The pain worsened with light touch and friction from clothing, prompting the patient to lose weight and avoid wearing a shirt while at work. The patient experienced minimal relief with pharmacologic and conservative measures. Intercostal injections did not improve his pain.

On February 3, 2021, the patient underwent CRFA of the left intercostal nerves at the levels of T9 and T10 under fluoroscopic guidance. Fifty-five days following the procedure, the patient's pain improved to 2/10 at best and 5/10 at worst. The sensitivity to light touch did not improve with the procedure. Since the 55-day post-CRFA follow-up, the patient has not reported a change in or return of pain.

## DISCUSSION

A summary of the 6 cases is provided in the Table. Because of the retrospective nature of this case series, no standardized methodology for reporting pain relief following the CRFA procedure was established. For patients who provided preprocedure and postprocedure VAS scores, we calculated their percent pain reductions from those 2 scores. The patients in cases 4 and 5 did not report a postprocedure VAS pain score but only provided a percent reduction. For these 2 patients, we used the percent reductions to calculate their postprocedure pain scores based on their preprocedure pain scores. The patient in case 5 reported pain as 5/10 with the worst as 10/10; for this patient, we used the preprocedure pain score of 10 for calculations. The patient in case 6 gave varying postprocedure pain scores; we averaged these scores to calculate a postprocedure pain score.

**Table.** Patient Demographics and Outcomes After Cooled Radiofrequency Ablation (CRFA)

Patient	Age	Sex	BMI	Imaging Findings	Level of CRFA	Preprocedure Pain Score^a^	Postprocedure Pain Score^a^	% Reduction in Pain Score
1	72	F	25.4	MRI: Multilevel degenerative disc disease with narrowing of the cervical spinal canal, scattered discogenic endplate changes, and small disc bulges at T7-T8 and T10-T11	Left T8-T11	9	0	100
2	31	F	21.3	X-ray: Very mild upper thoracic degenerative disc space narrowing	Right T7-T10	7	0	100
3	44	M	25.0	Not available	Right T1-T4	6	2	66.7
4	54	M	32.5	MRI: Chronic moderate compression of T12, minimal degenerative disc disease changes, slight desiccation of the disc at T2-T3 without bulge or hernia, mild disc space narrowing and a minor posterior central disc protrusion at T3-T4, and mild disc bulging at the C6-C7 level	Right T9-T12	9	1	88.9
5	46	F	20.9	X-ray: Nothing abnormal	Left T9-T12	10	2.5	75.0
6	57	M	29.7	Not available	Left T9 and T10	7	3.5	50.0
Average	50.7	–	25.8	–	–	8	1.5	81.3

^a^Pain was scored on a scale of 0 to 10, with 0 indicative of no pain and 10 indicative of maximal pain.

F, female; M, male; BMI, body mass index; MRI, magnetic resonance imaging.

All 6 patients who underwent CRFA to treat their intercostal neuralgia had tried one or more conventional treatments without lasting pain alleviation. The average age of the patients was 50.7 years, and the series included an even number of males and females. We used the average of the pain scores after the procedure to analyze the treatment effect. All 6 patients reported noteworthy pain reduction after CRFA, with an overall average pain reduction of 81.3%. Two patients, both female who presented with thoracic back pain that was not alleviated with NSAIDs and opioids, reported 100% pain relief posttreatment; their CRFA procedures ablated the lower thoracic nerves (T7-T10 and T8-T11).

The 2 female patients who experienced 100% pain relief had some key differences in their medical histories. One of the patients was 31 years old, while the other was 72 years. The 31-year-old patient had a minimal medical history beyond her intercostal neuralgia, while the 72-year-old patient had multiple comorbidities. These 2 cases suggest that CRFA for intercostal neuralgia is a potential treatment option for patients with various medical histories and wide age ranges. Common comorbidities were hypertension in 3 patients and depression in 2 patients. Other comorbidities ranged from osteoarthritis of the lumbar spine to migraine headaches.

To our knowledge, this case series is the first to examine the efficacy of CRFA for treating intercostal neuralgia. These 6 patients did not report any side effects related to the procedure other than typical postprocedure soreness that resolved within 2 weeks. After CRFA of the sacral lateral branch nerves, the incidence of postprocedure neuropathic pain is approximately 0.7% per lesion,^21^ so neuropathic pain can develop in other parts of the body. Infection, bleeding, needle placement–induced damage, and burns from grounding pad placement are all adverse effects of thermal lesioning; however, these side effects are rare.^22^ The prevalence of these side effects associated with CRFA treatment for intercostal neuralgia is not yet known.

The patient in case 3 had the least pain reduction. The patient had previously undergone conventional RFA to treat his intercostal neuralgia and experienced >70% reduction in his pain. His subsequent CRFA procedure resulted in a 66.7% reduction in pain. The decreased reduction in pain after CRFA compared to the original RFA may be attributed to having a second procedure. The efficacy of repeat CRFA can be variable, and patients who undergo 2 or more RFA treatments may have more or less relief after the subsequent treatments. A study assessing the efficacy of radiofrequency medial branch neurotomy for lumbar facet syndrome found similar pain relief with repeat radiofrequency neurotomy compared to the initial procedure.^23^ This topic needs further study, specifically for CRFA of the intercostal nerves.

Because of the retrospective nature of this study, postoperative pain assessments were completed after various intervals of time. Treatment plans for patients who undergo CRFA therapy and have a return of intercostal neuralgia symptoms are flexible, with the potential for CRFA therapy to be repeated if sustained pain relief was reported after the first treatment. Additional technologies could also be used, such as spinal cord stimulator and peripheral nerve stimulator therapies; however, treatment decisions require situational discretion and must align with the preference of the patient.

Patients with chronic pain have a lower quality of life compared to the general population,^24^ and intercostal neuralgia is not unreasonably rare. An efficacious treatment modality for intercostal neuralgia could be life-altering for many patients, especially those who are refractory to conservative measures.

Because this case series was not standardized, a long-term assessment of these 6 cases cannot be extrapolated. However, studies have demonstrated that CRFA treatment can provide pain relief from 70 to 250 days in the hip^25^ and up to 6 months in the knee,^26^ so pain relief for patients with intercostal neuralgia could potentially last as long. Further study should be conducted to assess the duration of effect for CRFA for the treatment of intercostal neuralgia specifically and to determine the side effects, ideal candidates, and safety.

## CONCLUSION

This case series shows that CRFA is a potentially favorable treatment for intercostal neuralgia, resulting in noteworthy reductions in pain. Our findings are consistent with CRFA studies for treating other neuropathies. To further elucidate the safety and efficacy of CRFA for treating intercostal neuralgia and to determine which patients would benefit the most from this treatment, large clinical studies must be conducted.

## Notes

*This article meets the Accreditation Council for Graduate Medical Education and the American Board of Medical Specialties Maintenance of Certification competencies for Patient Care and Medical Knowledge.*
